# Salivary and gingival crevicular fluid (GCF) protein biomarkers as proxies of root resorption following orthodontic tooth movement. A systematic review

**DOI:** 10.4317/jced.62494

**Published:** 2025-05-01

**Authors:** Jerry Kuruthukulangara, Theodore Eliades, Despina Koletsi

**Affiliations:** 1 Clinic of Orthodontics and Pediatric Dentistry, Center of Dental Medicine, University of Zurich, Switzerland; 2 Meta-Research Innovation Center at Stanford (METRICS), Stanford University, California, USA

## Abstract

**Background:**

Orthodontically induced root resorption (OIRR) is a common complication of orthodontic treatment, characterized by inflammatory and remodeling processes. This systematic review (SR) aimed to evaluate the potential of salivary and gingival crevicular fluid (GCF) biomarkers as proxies for the early detection of an ongoing root resorption process during orthodontic tooth movement.

**Material and Methods:**

Electronic searches were performed in multiple databases of published and grey literature, up to May 2023. Eligible studies included participants undergoing orthodontic treatment with reported biomarkers linked to root resorption against a control group. Analysis methods of the biomarkers such as ELISA, Western Blot, mass spectrometry, were utilized.

**Results:**

Seven studies met the inclusion criteria, with sample sizes ranging from 19 to 74 participants and varied study designs, with methodological heterogeneity. The detected proteins and biomarkers were dentin phosphoryn, dentin sialoprotein, dentin sialophosphoprotein, fetuin-A, IFN- γ, IL-10, IL-12p70, IL-7 p21-ARC, sIgA and these served as promising indicators of increased root resorption activity, during orthodontic treatment.

**Conclusions:**

Biomarkers and proteins in saliva and GCF demonstrated a potential for early detetction and monitoring of OIRR, offering a non-invasive alternative to radiographic techniques. However, standardization of protein and biomarker assessment protocols and development of large-scale studies seem imperative.

** Key words:**Biomarkers, gingivial crevicular fluid, orthodontic treatment, root resorption, saliva, systematic review.

## Introduction

External apical root resorption is a well- known, common and undesirable complication of orthodontic treatment (1). It has been reported even in more than 50 per cent of orthodontic patients treated with fixed appliances, albeit the majority of cases are affected in a limited amount and thus there is no compromise in the dental functions and tissues (2). The resorption process entails the removal of the hyaline bone zone, during which the adjacent outer layer of the root cementum is harmed. Concurrently, osteoblasts are engaged in the reparative process at the outmost and interfacial area between the bone and the root surface, persisting until there is either no hyaline tissue left or the applied force is interrupted (3).

Early detection of root resorption during orthodontic treatment plays a pivotal role in identifying teeth at risk of severe resorption. Currently, the detection of root resoprtion relies largely on radiographic techniques. However, conventional radiographs may detect resorption once approximately 60-70% of the mineralized tissue has been lost, and primarily offer two-dimensional information, mainly identifying changes at the apex. It has been suggested that periapical radiographs do not reveal orthodontically induced root resorption regions, which have been histologically verified, even after seven weeks of treatment (4-5). Furthermore, radiographs cannot confirm whether the process of root resorption is still ongoing, while monitoring the progression of root resorption necessitates additional radiation exposure for the patient.

The fundamental basis of orthodontic tooth movement comprises micro- trauma within the periodontal ligament, which triggers a localized cycle of periodontal inflammation (6). The latter leads to the production and secretion of various cytokines in the local area, some of which promote bone resorption (pro-resorptive) while others inhibit the process (anti-resorptive) (7-9). As such, the existence of protein markers within the gingival crevicular fluid (GCF), notably for example dentin matrix protein (DMP-1), has been established in individuals undergoing orthodontic treatment. Since then, there has been substantial progress in the assessment of the role of these proteins, and research exploring their linking with resorption has substantially developed (10).

Among the pro-resorptive cytokines one may identify the members of the interleukin-1 (IL-1) family, such as IL-1β, IL-6, IL-7, IL-8, and TNF-α. IL-1β directly stimulates the formation and function of osteoclasts, cells responsible for bone resorption. Similarly, IL-6 acts in synergy with IL-1 and TNF-α to enhance osteoclast formation and function (11,12). IL-7 is also indirectly implicated by inducing TNF-α, which plays a vital role in promoting receptor activator of nuclear factor kappa-B ligand (RANKL)-mediated osteoclastogenesis (13). IL-8, increases the expression of RANKL, thereby boosting osteoclast generation and activation (14).

Conversely, anti-resorptive cytokines like IL-4 and interferon-gamma (IFN-γ) inhibit osteoclastogenesis and T-cell-mediated RANKL-induced osteoclast formation, respectively (15). Granulocyte macrophage colony-stimulating factor (GM-CSF) is another anti-resorptive cytokine that, alongside IL-4, IL-10, IL-13, IL-18, and IFN-γ, works synergistically to suppress bone resorption (16,17).

Given the key role of saliva and gingival crevicular fluid as non- invasive diagnostic tools, their contribution to early detection of one of the most common orthodontic tooth movement complications is apparently of utmost significance for both the clinician and the patient. There is no similar attempt, to date, to collect and appraise all available evidence on the role of salivary/GCF protein molecules regarding orthodontically induced root resorption. Therefore, the purpose of this systematic review was to identify, summarize and critically appraise all relevant biomarkers and proteins which might indicate the presence of an ongoing root resorption process during orthodontic tooth movement.

## Material and Methods

-Protocol and Registration

The study protocol was drafted and apriori registered at the Open Science Framework (link: https://osf.io/26hz3/). The review has been reported according to PRISMA 2020 reporting guidelines (18).

-Search strategy

An electronic search was conducted on December 14, 2020 and updated on May, 2023, within published and unpublished literature, separately and by two examiners. The databases sought since inception were: Pubmed via MEDLINE, EMBASE, the Cochrane Database for Systematic Reviews (CDSR), the Clinicaltrials.gov, the ISRCTN registry, and the Open Grey.

The search strategy is presented below:

((salivary biomarkers) OR (crevicular fluid biomarker) OR (saliva* biomarker*) OR (peptide*) OR (salivary protein) OR (protein biomarker) OR (saliva cytokine*) OR (saliva interleukin)) AND ((orthodontic treatment) OR (orthodontic) OR (tooth movement)) AND ((root resorption) OR (orthodontically induced inflammatory root resorption) OR (OIIRR) OR (orthodontically induced external apical root resorption) or (OIEARR))

-Eligibility criteria

Inclusion:

Study Design: any study design, observational (cohort, case control, cross- sectional) or interventional (RCT, clinical trial)

Population: human, of any age/ sex

Condition: orthodontic tooth movement

Exposure: biomarkers/ proteins (any) detected in saliva and/ or gingival crevicular fluid related to root resorption during tooth movement

Outcome: root resorption

Exclusion: studies other than in human (ie, animal studies, *in vitro*)

-Study Selection

Two reviewers independently evaluated the titles and abstracts of the articles initially retrieved. In the second stage, both reviewers assessed the full text of potentially eligible articles and decided on finally included papers.

-Data collection process

Data was extracted and documented according to the various study attributes, such as study design, title, authors, publication date, population details, condition, exposure, and outcomes. Two reviewers conducted the data extraction, and a third reviewer subsequently reviewed the extracted data. Any inconsistances were identified and resolved through discussion until a consensus was reached.

-Risk of bias in individual studies (internal validity)

Risk of bias assessment of individual studies was based on their study design. For RCTs (if any), the Cochrane RoB tool 2.0 was used (19). For non- randomized prospective controlled trials or observational studies, the ROBINS-I tool was used (Risk of Bias in Non-randomized Studies of Interventions) (20).

-Quantitative synthesis

If applicable and after examining levels of heterogeneity across individual studies, quantitative synthesis/es was planned to be conducted. In view of the variability in treatment procedures and settings across studies, random effects meta – analysis would be conducted if applicable. If 2 or more studies were considered eligible, pooled estimates would be be presented. Statistical heterogeneity would be further explored through an I-squared test, where a *p-value* below the level of 10% (i.e, *p*<0.10) is indicative for non- homogeneity. Visual inspection of heterogeneity was also be based on confidence interval (CI) overlap within the constructed forest-plots. An estimate of between study variance would be given as tau- squared (τ2). Effect measures used were mean differences (MD) for continuous outcomes retrieved and odds ratios (ORs) for dichotomous (if any). In both cases, measures of precision would also be reported (95% confidence intervals, CIs), if possible.

-Publication bias

Publication bias was planned to be explored through standard funnel plots and Egger’s regression test, conditional on the inclusion of more than 10 studies in the quantitative synthesis (21). However, due to the limited number of studies eligible, its exploration was not applicable.

## Results

-Search details

**Figure 1:**
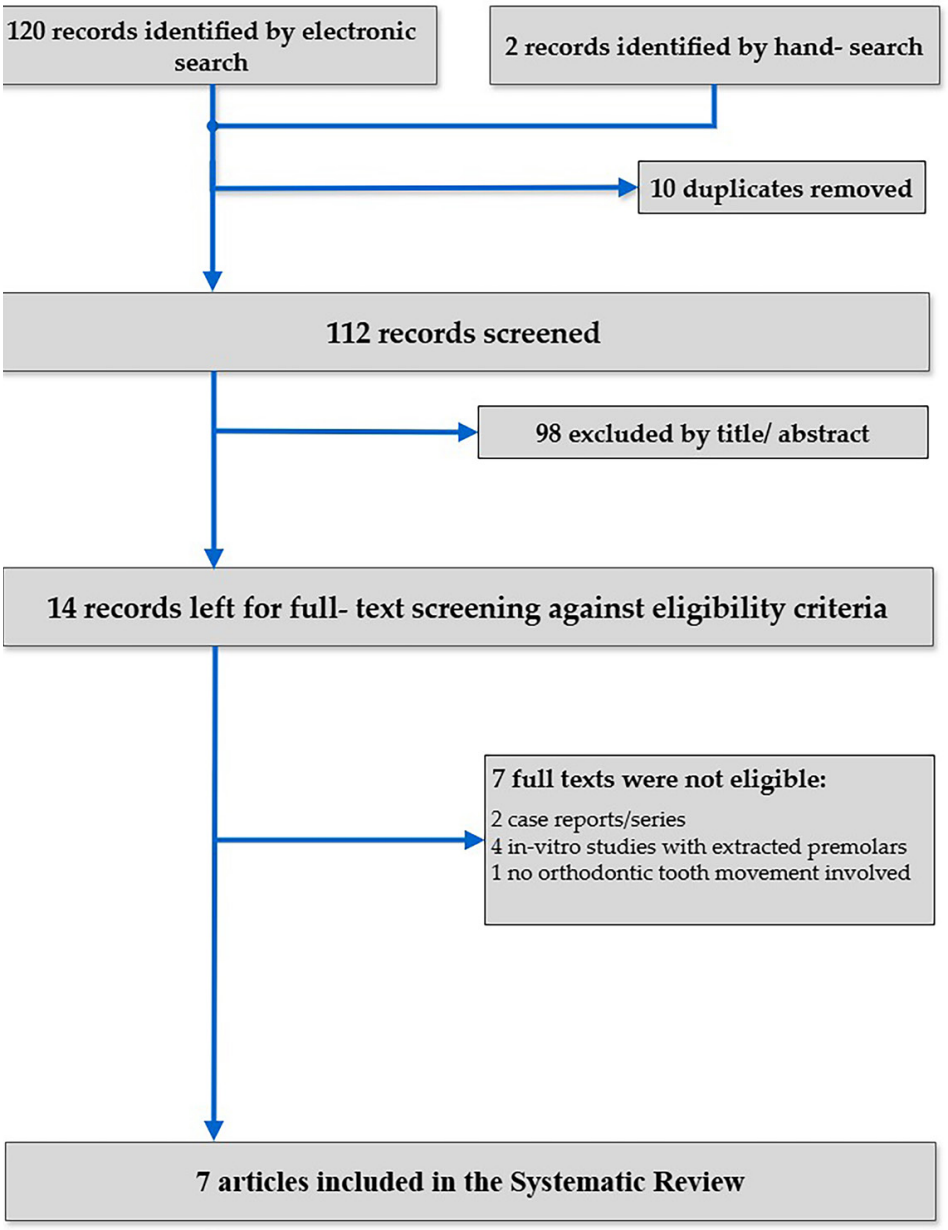
Flowchart of study selection and inclusion.

From an initial number of 122 results, 14 studies passed through full-text evaluation. Ultimately, 7 studies were considered suitable for inclusion in the systematic review (Fig. 1).

Study design and characteristics

Three of the studies were case-control (22-24), three were designed as cross- sectional (25-27). The remaining one was a cohort study (28). The design, exposure, condition and outcome of each study are summarized in detail in  Table 1.

In summary, the sample sizes across the studies ranged from 19 to 74 patients, with age range varying from 10 to a maximum of 44 years old. Zhou *et al*., 2017 (27), only included female patients. Treatment length was at least six months long in the study of Ramos *et al*., 2011 (28), 9 months in one study (23) and another one extended up to two years (24). The rest studies (22,25-27) did not report on treatment duration. Regarding the treatment modalities used during treatment, Ramos *et al*., 2011 (28) and Zhou *et al*., 2017 (27), reported the utilization of fixed appliances with straight-wire technique, while the other studies (22,24-26) did not specify on the orthodontic methods and devices used during treatment. The assessment method of root resorption varied across the included studies. Mandour *et al*., 2021 (25) and Zhou *et al*., 2017 (27) performed panoramic radiographic assessments to measure root resorption while the other five studies (22-24,26,28) reported the use of periapical radiographic imaging.

Regarding biological samples, three out of the seven studies evaluated gingival crevicular fluid as the primary biomarker source (22,25-26). Two studies focused on samples of saliva (23,27) and two studies assessed additional to saliva, blood samples of the patients (24,28). Zhou *et al*., 2017 (27), is the sole study that used H-NMR analysis. In contrast, Balducci *et al*., 2007 (22), utilized a combination of SDS-PAGE, Western Blot, and ELISA to investigate dentin-specific proteins in GCF. Similarly, Kaczor *et al*., 2017 (23), applied quantitative mass spectrometry (qMS), two-dimensional gel electrophoresis and Western Blotting to identify salivary protein biomarkers associated with orthodontically induced root resorption (OIRR). Mandour *et al*., 2021 (25), and Ramos *et al*., 2011 (28), relied on ELISA. Vieira *et al*., 2014 (26), applied SDS-PAGE while Yashin *et al*., 2017 (24), utilised multiplex ELISA arrays.

In terms of the outcome variable and its proxies assessed, while four studies (22-23,25-26) examined proteins as potential root resorption indicator, the other three (24,27-28) focused more on Interleukin and other cytokines to indicate inflammation or cellular responses related to root resorption.

-Effects of Interventions, and within study analyses 

The selected studies differ in their approach of acquiring the type of sample, analytical instruments and methods of measuring root resorption. Albeit we anticipated that it would be possible to synthesize data from several studies, there was no further information regarding inadequately reported data, or raw data from all but one study (28). Efforts were made to obtain raw and individual participant data from all studies, through direct email requests and follow-up messages to the corresponding authors, but no responses were received. There was a wide variation of methodologies followed across the included studies and as such, the main findings of the included studies are presented below in summary.

Balducci *et al*., 2007 (22), identified dentin phosphophoryn (PP) and dentin sialoprotein (DSP) as potential indicators for root resorption, which are both non-collagenous dentin matrix proteins involved in dentin mineralisation and typically released during the active process of root resorption. Their concentrations were significantly higher in severe root resorption cases than in mild cases or the control/ no tooth movement- no resorption group. The control group showed the lowest levels of PP and DSP. Kaczor *et al*., 2017 (23), highlighted fetuin-A (a secreted plasma protein) and P21-ARC (an amino acid actin-binding component of actin related protein 2/3 complex (Arp2/3)) as potential biomarkers with increased expression in the saliva of patients with OIRR. Utilisation of two-dimensional gel electrophoresis and mass spectrometry to detect proteins in saliva showed an average increase of 1.3 fold of p21-ARC and average increase of 1.2 fold of fetuin-A in moderate to severe resorption groups. Mandour *et al*., 2021 (25), focused on dentin sialophosphoprotein (DSPP) and Interleukin-1 receptor antagonist (IL-1ra). DSPP showed higher concentration levels in the GCF in root resorption cases with a diagnostic cut off of 7.33 pg/ml while IL-1ra concentration levels were lower in cases of root resorption compared to the control/ no tooth movement- no resorption group with a cutoff of 432.6 pg/ml. Ramos *et al*., 2011 (28), focused on the association of salivary secretory IgA (sIgA) and serum IgG (sIgG) antibodies against dentin matrix proteins with root resorption. Patients who developed moderate to severe root resorption 6 months after treatment, presented increased sIgA levels at the initiation of treatment (mean sIgA, 0.434; standard deviation (SD), 0.203) in comparison with those who developed slight resorption (mean sIgA, 0.188; SD, 0.095) or no resorption (mean sIgA, 0.136; SD, 0.051). The analysis of serum IgG in the blood did not correlate with lesion severity. Vieira *et al*., 2014 (26), found dentin matrix protein (DMP-1), dentin phosphoprotein (DPP) and dentin sialoprotein (DSP) as possible indicators for root resorption in the GCF. Higher concentrations of DPP and DSP were observed in severe cases of root resorption compared to mild and control/ no resorption groups. Yashin *et al*., 2017 (24), reported decreased cytokine levels of IL-4 and increased levels of IFN-γ, IL-7, IL-10, and IL-12p70 to be associated with root resorption in saliva samples and higher levels of osteocalcin and procollagen type I N-terminal peptide (P1NP) in blood samples of the control/ no resorption group. No significant difference for the aforementioned biomarkers was identified between moderate and severe root resorption. Zhou *et al*., 2017 (27), reported a range of metabolic biomarkers, including butyrate, propane-1,2-diol, α-linolenic acid, α-glucose, urea, fumarate, formate, guanosine, and purine. Elevated levels of metabolites such as butyrate and purines were found in saliva samples of patients with OIEARR.

Risk of bias within studies 

**Figure 2:**
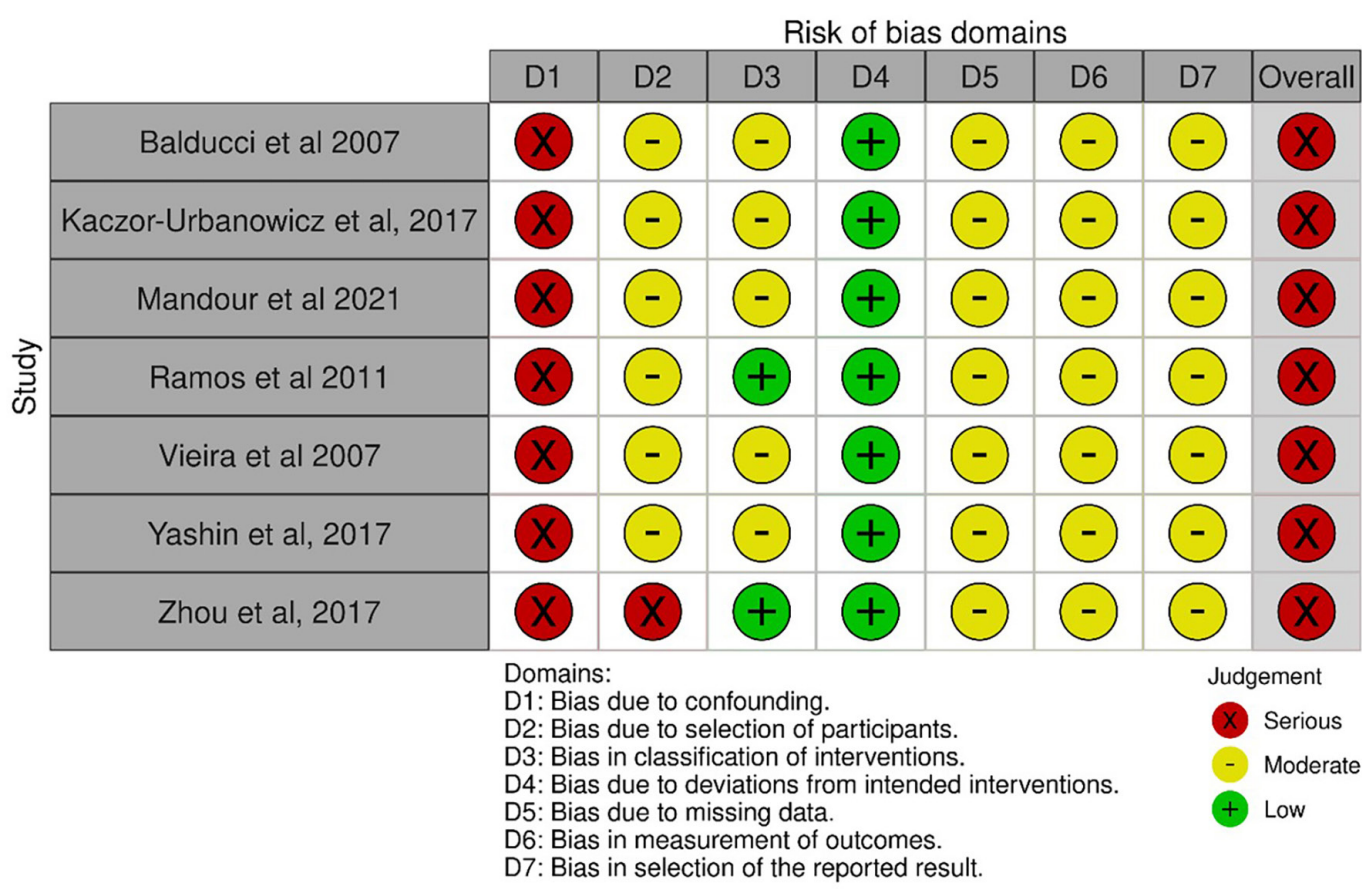
Risk of bias summary graph across studies [ROBINS-I tool for non-Randomized Prospective Clinicial Trials, nR-PCTs].

All studies received a rating of serious overall risk of bias. All studies received a serious RoB rating for confounding, and a moderate RoB rating for selection bias except for Zhou *et al*., 2017 (27), which rated as having a serious risk. All studies were marked with a moderate risk assessment for classification of interventions/exposure bias, except for Ramos *et al*., 2011 (28), and Zhou *et al*., 2017 (27), which were rated as low risk. All studies were assigned a moderate risk of bias due to missing data, measurement of outcomes and selection of the reported result. Regarding deviations from intended interventions, there was evidence of low RoB for all studies (Fig. 2) (Supplement 1) (http://www.medicinaoral.com/medoralfree01/aop/jced_62494_s01.pdf).

## Discussion

-Findings in context

Root resorption is a common but undesirable outcome of orthodontic treatment, involving the breakdown of root structures due to inappropriately employed mechanical forces and conditional on genetic predisposition. In this SR our findings highlight the existing but limited evidence supporting the potential of biomarkers and proteins in saliva, gingival crevicular fluid (GCF) and additionally blood, to contribute to the early diagnosis of root resorption associated with orthodontic treatment.

The state of the art regarding proxy biomarkers for early identification of root resorption following orthodontic tooth movement is currently framed by evidence prone to serious risk of bias overall. This suggests that the findings should be interpreted with caution and acknowledgement of all emerging caveats. Participants and their treatment or the selection of control did not follow a randomization scheme, thus, selection bias or risk for confounding might be likely. Pre-existing dental conditions, undiagnosed systemic diseases, small sample sizes and lack of age- based interpretation of the findings or exploration of interaction including sex and age, is likely to introduce bias in the reporting and interpretation of the results of the included studies.

Limited information is available regarding the specifics of the orthodontic treatment and its duration, following which the identified root resorption was developed. Crucial details such the type of wires used with their corresponding applied forces, the duration of the forces, or other factors identified by the literature as predisposing for the development of root resorption remain unspecified. Additionally, it appeared that no long-term follow-up to assessment of the extent of root resorption was recorded, which apparently may occur following active orthodontic treatment, within the posttreatment period.

There has been early evidence of genetic predisposition to external apical root resorption in orthodontically treated patients, which has been recorded as independent of age, sex or severity of malocclusion, but documented with reduced variance within siblings (29). Additionally, the extent of root movement within the bone apparatus, and the presence of long, narrow and deviated roots with pointed or deviated apices have been recorded as risk factors for apical root resorption (30). There further seems to exist a positive correlation between higher force levels, longer treatment duration, and an increase in root resorption. A pause in tooth movement has appeared to be an advantageous protective measure in minimizing root resorption, as it provides time for the resorbed cementum to recover (31). Yassir *et al*., 2021 (32), adds also that heavy and continuous forces with fixed appliance treatment, and types of orthodontic movement such as intrusion, and/ or retraction are factors which may also lead to a higher risk of OIIRR.

Furthermore, the evidence stemming from the present review relates only to outcome assessment based on panoramic or periapical radiographs used as imaging tools. These provide only a two-dimensional representation of the tooth and the surrounding structures. Specifically, OPGs are subject to distortion and as such compromise the reading of the size of the tooth and subsequently the defect. Periapical radiographs, while offering greater detail, pose challenges in reproducibility, since it is utterly difficult to obtain identical before- after pairs of radiographs of a tooth structure even when a sole clinician is involved.

Despite the inability to conduct a meta-analysis due to limited data that could be used statistically or non- provision of raw data/ individual participant data by the authors of the included studies (missing standard deviations and standard errors), we observed qualitative patterns suggesting the role of several biomarkers in the inflammation and resorption processes, which however, needs further exploration. The clinical potential of using biomarkers, particularly in saliva and GCF as non- invasive means for the early detection of orthodontically induced root resorption could reduce dependency on radiography. The latter often detects resorption only after significant substance loss has occurred and the defect is impossible to observe or diagnose at the time the process is actively ongoing. Using biomarkers as a non-invasive diagnostic method could lead orthodontists to actively adopt a point- of- care philosophy that might assist them in adjusting forces or treatment protocols in resorption-prone individuals, during the active course of treatment (33).

-Strengths and Limitations

The strengths of this review include its thorough search strategy, systematic approach and exploration of bias, ensuring an examination of potential biomarkers associated and serving as proxies to root resorption. Promising potential indicators were associated with root resorption in these studies through reliable measurement techniques such as ELISA. The use of NMR spectroscopy broadens the potential indicator for root resorption with a comprehensive metabolic profile. The extraction of different body fluids, non-invasively, such as saliva and GCF, provides more insight into the process of root resorption and its influential factors.

However, the inability to conduct a quantitative synthesis or additional analyses, due to limited data and/ or no- response by the authors of the included studies restricts the strength and precision of the interpretation of the findings and the conclusions. Several shortcomings associated inherently with the available information compromises the level of evidence provided. Small sample sizes, type of diagnostic ascertainment of the outcome of root resorption, wide variation in the approaches utilized to define potential proxies, missing statistical data, orthodontic treatment details including follow- up duration, and non-standardized treatment protocols of treatment, global lack of individual study registration of the protocol risking selective reporting, may all reduce the validity of the findings and compromise future reproducibility related to this field of research.

## Conclusions

The potential of saliva and GCF-specific biomarkers to serve as proxies and detect root resorption during orthodontic treatment is highlighted in this review. While the findings are promising, the absence of consistent quantitative data emphasizes the need for methodological improvements. Upcoming research should prioritize studies with standardized biomarker assessment and reporting protocols to enable a robust quantitative synthesis of evidence and provide a clinical approach for the improvement of point- of- care diagnostic strategies in the OIRR monitoring.

## Figures and Tables

**Table 1:** Characteristics of included studies.

Study	Population	Exposure	Condition	Outcome	Notes
Balducci et al., 2007 Case-control study Biological Markers for Evaluation of Root Resorption	60 divided into three groups of 20 each: Mild resorption (<2mm) Severe (>2mm) Control Control: 13f & 7m, age range 12-34y Mild: 11f & 9m, age range 14-40y Severe: 15f & 5m, age range: 16-44	Gingiva crevicular fluid mesial & distal side of upper central and lateral incisor SDS-polyacrylamid gel electrophorese Western blotting: antibodies against DMP1, PP and DSP ELISA	orthodontic therapy for mild and severe group control has not started orthodontic therapy yet	Dentin matrix protein 1 (DMP1) statistically significant difference control to study groups but none to between mild and severe Dentin phosphophoryn (PP) statistically significant higher concentration in severe then mild then control Dentin sialoprotein (DSP) severe statistical significant higher concentration than mild, control has the least PP and DSP could be potential marker for root resorption because their concentration are higher than in control groups. DMP1 presence seems not only be the result of resorption activity but also because of increased bone remodeling during orthodontic tooth movement, so it is not a good marker because it is not possible to distinguish between normal and pathologic activity	Orthodontic therapy for at least one year and with radiographic signs of root resorption
Kaczor-Urbanowicz et al, 2017. Case-control study Identification of salivary protein biomarkers for orthodontically induced inflammatory root resorption.	72 subjects 48 (31f & 17m) Orthodontically induced inflammatory root resorption (OIIRR) 24 (13f & 11m) untreated Range 10-30y	Whole saliva (WS) Removal of albumin and igG through Proteopre immunoaffinity albumin and igG depletion kit SDS-PAGE Western Blotting	Division by age and root resorption → 6 groups age 10-20 = young and 21-30 = adult moderate-to-severe RR, apically ≥ 2mm mild RR apically < 2mm control, no RR apically Moderate-to-severe root resorption (RR) 11f & 6m Moderate-to severe RR young → 7f & 4m Moderate-to severe RR adult → 6f & 3m Mild RR young →7f & 4m Mild RR adult → 6f & 3m Control young → 7f & 6m Control adult → 6f & 5m Young patients with severe root resorption was associated with actin cytoskeleton regulation and/or FC gamma R-mediated phagocytosis In adult patients it was related to focal adhesion Moderate-to-severe young group: the functional cluster of proteins are assiociated with mainly acute inflammation/response to wounding/defense response Whereas in adults is more related to chronic/programmed processes (apoptosis or focal adhesion) P21 ARC and fetuin-A selected as candidate biomarkers for further validation based on their known association with inflammation, hard tissue resorption and orthodontic tooth movement. However those protein have not previously been identified in human saliva	Radiographic assessment of pericapical x-rays of four upper incisors taken before and 9 months after bonding was done Proposing fetuin-A and p21-ARC as biomarker candidates for OIIRR
Mandour et al., 2021 Cross sectional study Expression of biological markers in gingival crevicular fluid of teeth with orthodontically induced root resorption	74 patients in 3 groups: Orthodontic 25 (15f, 10m) showed 1-3mm root resorption of a maxillary central incisor Pediatric 24 (12f, 12m) root resorption of a lower second primary molar by loss of about half the roots with the presence of the successor control groups 25 (19f, 6m) no resorption	Gingival crevicular fluid of the maxillary central incisor ELISA	Orthodontic group had treatment for over a year	Significant differences in the levels of Interleukin-1-receptor antagonist (IL-1ra) and dentin sialophosphoprotein (DSPP) between the three groups. IL-1ra levels highest in control group, followed by the orthodontic group and then by the pediatric group DSPP levels highest in the pediatric group, followed by the orthodontic group and lowest in the control group. DSPP: Suggestion of possible association with OIRR	Sensitivy and specificity of IL-1ra for the diagnosis of OIRR showed 80% reliability Sensitivity and specificity of DPP for the diagnosis of OIRR showed 100% reliability Limitations: notes the use of 2D radiographs may not provide precise information about the extent and nature of resorption.
Ramos et al., 2011 Cohort Study Anti-dentine antibodies with root resorption during orthodontic treatment.	50 orthodontic individuals selected 19m (15.6 ± 8.5y) & 31f (21.4 ± 11.2y) 19 in the mixed dentition (10.3 ± 1.9) 31 in permanent dentition (24.6 ± 9.9y) 50 individuals not undergoing orthodontic treatment were selected as control group. 36 f (26.4 8.1y) 14 m (17.6 ± 10.0y) 24 mixed dentition (9.4 ± 1.2y) and 26 in the permanent dentition (23.5 ± 6.4y)	Saliva and blood taken at T0 and T6 Serum samples collected from the permanent dentition group (n = 31)(ethical reasons). Serum IgG levels and salivatory secreatory IgA (sIgA) levels in human dentin extract (HDE) at T0 and T6 ELISA	Edgewise or straightwire fixed orthodontic appliances	At T6 rooth resorption was classified as Grade 0 (no resporption) (n= 18) Grade 1 (slight resorption) n= 24 Grade 2 (moderate to severe resorption) N = 8 Degree of root resorption was associated with age and the presence of abnormal root shape igG levels in control group did not differ from patietns at T0. The mean difference in patients' IgG levels from T0 to T6 was not significant, except for the grade 2 root resorption group. No association between degree of root resorption and igG levels at T0 and T6 in the permanent dentition. Anti-HDE sIgA levels did not differ between patients or controls at T0. Patients' sIgA levels at T0 did not differ from those at T6. Patients who developed grade 2 root resorption at T6 presented increased sIgA levels at T0 in comparison with grade 1 and grade 0. Increased sIgA levels in saliva in the beginning of the therapy in patients who later showed moderate to severe resorption after 6 months of treatment Serum IgG anti-HDE during orthodontic treatment does not correlate with lesion severity but may help to explain some of the immunopathological mechanism of the process.	Radiograph of upper central incisor at T0 and T6 Salivary sIgA levels may serve as a potential marker for identifying patients at risk of root resorption before treatment.
Vieira et al., 2014 Cross sectional study Protein biomarkers of external root resorption: a new protein extraction protocol. Are we going in the right direction?	60 patients 38 female 22 male Age (15-30y) Group 1: control, 30 patients who had been undergoing treatment for atleast 6 months without inflammatory external root resorption (IERR) Group 2: 30 patients, orthodontic treatment for at least 6 months with mild to moderate IERR	Gingival Crevicular Fluid SDS PAGE Gel electrophoresis	Orthodontic treatment for at least 6 months	Discussion about Balducci: DMP-1 not good root resorption marker, since it doesn't allow us to distinguish between normal and pathological activity Balducci et al studied PP and DSP proteins, and found larger concentrations of PP an DSP in the severe IERR group, followed by the mild IERR group and the control group. However, the authors used polyclonal antibodies that react to proteins with similar epitopes and may indicate the presence of small amounts of DPP and DSP in the control group. The literature does not reach a consensus regarding DPP and DSP proteins as molecular markers due to the presence of these proteins in control groups (even in small quantities) Further high-resolution protein research methods searching for new molecular markers are still necessary. Two-dimensional electrophoresis followed by mass spectrometry is the technique of choice for this task	The gel performing protein extraction by means of sodium phosphate buffer solution showed traces of salt and did not achieve the first dimension of isoelectric focusing in the two-dimensional electrophoresis Cementum proteins may be disqualified as IERR markers because during orthodontic movement cementum is reabsorbed and subsequently repaired. Cementum degradation in the GCF were detected by most researches in both control and treatment groups Referral to Balducci et al about DMP1, DPP and DSP in GCF
Yashin et al., 2017 Retrospective Case Control Study Markers in blood and saliva for prediction of orthodontically induced inflammatory root resorption: a retrospective case controlled-study.	9 patients 6 female 3 male Age: 23 ± 2.9y M: 25 ± 3.6y F: 22 ± 2.2y 3 of those 9 have moderate OIIRR and the remaining had severe orthodontically induced inflammatory root resorption (OIIRR) 16 (4f & 12m) patients who have not developed OIIRR were used as control	Blood and saliva Fasting 12h before blood and saliva collection ELISA	Finished orthodontic treatment within 2 years 2010 - 2012 and not receiving any active orthodontic treatment at the time of study	Statistically significant decrease in cytokine expression of Interleukin 4 (IL-4) Statistically significant increase in cytokine expression of IFN- γ, IL-10, IL-12p70 and IL-7 in the root resorption group (RRG) than in the control group (CG) But no significant difference in moderate and severe RRG itself. Osteocalcin and P1NP only blood factor with significant difference between CG and RRG but gender has more effect on those two than diagnosis. These two do not seem to be useful markers. Cytokine expression between different diagnostic groups is more sensitive using saliva than in blood samples. Expression of cytokines may also be affected by any other potential inflammation in the body. Sample size is still too small to define link between cytokines in OIIRR. Recommendation of microfluidic biosensors.	Following index for root resorption in 1988 paper of Lavender and Malmgren: Grade 1: irregular root contour Grade 2: apical root resorption < 2mm Grade 3: apical root resorption 1/3 of original root length > X > 2mm Grade 4: apical root resorption > 1/3 of original rooth length Orthopantomograms OIIRR = (r1-r2) * (C1/C2) C1/C2 = magnification error R1-r2 = radiographical root length pretreatment minus post-treatment Limited in its capacity to account for tiliting of teeth especially maxillary and mandibular anterior teeth which are the most commonly affected teeth with extensive OIIRR.
Zhou J. 2017 Cross-sectional study A pilot study of the metabolomic profiles of saliva from female orthodontic patients with external apical root resorption.	Only adult female patients Age range 18-35y with orthodontic treatment 19 participants 8 EARR patients 11 controls	Saliva H NMR analysis (nuclear magnetic resonance spectroscopy)	Straight-wire technique fixed orthodontic treatment, no orthodontic retreatment	Results show that HNMR based metabolomics method can detect well different metabolites in saliva from OIEARR. Some characteristic metabolites inform us it relates inflammation and energy metabolism in oral microenvironment. Butyrate, Propane-1,2-diol, α-Linolenic acid, α-glucose, urea, fumarate, formate, guanosin, purine and others. Arachidonic acid metabolites have been shown to be related to PDL damage caused by oxidative stress, as consistent with the inflammatory responses in the PDL while OIEARR. Besides, purine metabolism which has been reported to be associated with the chemotaxis of inflammatory cells also indicates an increased inflammatory response. V.M Barnes demonstrated that the purine degradation pathway was accelerated in periodontitis. These results may suggest that the inflammation resulted from OIEARR is somewhat like the PDL breakdown in periodontitis converging in this purine pathway via oxidative stress. Nevertheless, this idea is speculative, and need to be more fully evaluated.	OPG before (T1) and after treatment (T2)

## Data Availability

Data underlying this study are available from the corresponding author upon request.
